# The influence of pulsed electric fields and microwave pretreatments on some selected physicochemical properties of oil extracted from black cumin seed

**DOI:** 10.1002/fsn3.535

**Published:** 2017-11-20

**Authors:** Hamid Bakhshabadi, HabibOllah Mirzaei, Alireza Ghodsvali, Seid Mahdi Jafari, Aman Mohammad Ziaiifar

**Affiliations:** ^1^ Department of Food Materials and Process Design Engineering Gorgan University of Agricultural Sciences and Natural Resources Gorgan Iran; ^2^ Agricultural Engineering Research Department Golestan Agricultural and Natural Resources Research and Education Center AREEO Gorgan Iran

**Keywords:** Black cumin seed, efficiency, microwave, oil extraction, oxidative stability, pulsed electric fields

## Abstract

Application of novel technologies such as microwave and pulsed electric fields (PEF) might increase the speed and efficiency of oil extraction. In the present research, PEF (3.25 kV/cm electric field intensity and 30 pulse number) and microwave (540 W for 180 s) pretreatments were used to study the process of oil extraction from black cumin (*Nigella sativa*) seeds. After applying the selected pretreatments, the oil of seeds was extracted with the use of a screw press and the extraction efficiency, refractive index, oil density, color index, oxidative stability, and chemical components of oil and protein of meal were evaluated. The achieved results expressed that PEF and microwave pretreatments increased the oil extraction efficiency and its oxidative stability. Different pretreatments didn't have any significant influence on the refractive index of black cumin seed oil (*p*>.05). When microwave and PEF were used, the oil density showed an enhancement as the following: 1.51% and 0.96%, respectively in comparison with the samples with no pretreatments. Evaluation of the extracted oils, using GC/MS analysis indicated that thymoquinone was the dominant phenolic component in the black cumin oil. Finally, the SEM analysis revealed that microwave and PEF can be useful in the extraction of oil from black cumin seeds since these treatments damaged cell walls and facilitated the oil extraction process.

## 1 INTRODUCTION

Oils and fats are important nutrients in the case of health and also in business. Due to these reasons, much research and investment are allocated to study them. Oils and fats are responsible to supply a significant part of required energy for body, necessary fatty acids, and fat‐soluble vitamins (Kebriti, HoseiniMazhari, Gerami, Ghiassi, & Esfandyari, 2011). Recently, with the growth and increase in general knowledge, the demand for health‐promoting oils in addition to energy supply increased. On the other hand, utilization of available sources and cultivation of underexplored oilseeds is a step toward providing more functional oils in the food industry. Oils and fats are prepared from various plant and animal sources of which have different metabolic, physical and chemical properties (Zomorrodi, Shokrani, Shahedi, & Dokhani, 2003). Black cumin (*Nigella sativa L*.) variety is an annual herbaceous with a short‐life term, that is, particular to the semiarid areas. Thymoquinone is detected as one of the main phenolic components of black cumin with antioxidant properties (Al‐Othman, Ahmad, Al‐Orf, Al‐Murshed, & Arif, 2006; Yar, El‐Hariri, El‐Bahai, & Bamosa, 2008). The antioxidant compounds have shown their activities via different mechanisms (Farzaneh & Carvalho, 2015).

There are various conventional methods to extract oil from oilseeds such as extraction with solvents through various extractors or extraction by mechanical approach such as pressing. Generally, oil extraction with pressing is a simple, safe and economical technology; therefore the advantages of this method makes it more efficient compared to solvent application approach. The mechanical method is used for oilseeds with high oil content. Applying this method solely is not sufficient and high amounts of oil remain in the meal which should be extracted by solvents. Oil extraction by pressing is done in hot or cold conditions. The yield of oil extracted in hot pressing is higher than cold pressing but because of elevated temperatures during hot pressing, the quality of the obtained oil is low while the oil obtained through cold pressing maintains its natural properties with limited chemical reactions. For this reason, the demand for oils obtained from cold pressing increased (Singer, Nogala‐Kalucka, & Lampart‐Szczap, 2008). In oil extraction with cold pressing, several factors such as pressing pressure, seed moisture and process temperature, should be considered in the efficiency of extracted oil.

In modern technologies, it is tried to perform the extraction process more easily and effectively in such a manner of solvent usage, extraction time and temperature could be reduced while the extraction efficiency can be improved economically (Li, Pordesimo, & Weiss, 2004). In the oil extraction process, appropriate pretreatments of seeds before oil extraction is one of the most important and necessary steps to produce a high‐ quality oil with a high efficiency (Azadmard‐Damirchi, Habibi, Hesari, Nemati, & Fathi, 2010). Therefore, recently some techniques such as extraction with supercritical solvents (Meireles & Angela, 2003), ultrasound (Dolatabadi et al., 2016; Jalili, Jafari, Emam‐Djomeh, Malekjani, & Farzaneh, 2017; Lou, Wang, Zhang, & Wang, 2010), microwave pretreatments (Bakhshabadi et al., 2017), and pulsed electric fields (Zeng, Han, & Zi, 2010) have been investigated.

The microwave, nonionized electromagnetic waves with 300 MHz to 300 GHz frequency are placed between radio and infrared waves in the electromagnetic spectrum. These waves are made of two oscillating vertical fields, which are electrical and magnetic fields. In this procedure, heating is occurred in a determined and chosen procedure without temperature waste and dissipation in the environment, it means that it is similar to heating in a closed system. This heating mechanism leads to a lower extraction time in comparison with common and usual oil extraction methods. The influence of this procedure is through two phenomena of ionic transfer and dipolar rotation and mostly, both of them are occurred simultaneously (Mandal, Mohan, & Hemalatha, 2007). The energy of microwave penetrates into the food and generates internal temperatures, leading to a more heating rate and shorter procedure time. The oilseeds have high moisture content as a dipolar material, although in this case some other materials, including salts and proteins can also act as the dielectric components (Sultana, Anwar, & Przybylski, 2007). The procedure of pulsed electric fields (PEF) has more advantages toward, using the common heating procedures for foods, because its application leads to maintain the original color, aroma and taste, texture and nutritional value of foods. Furthermore, PEF might increase the speed and efficiency of the extraction. PEF has been used in deactivation of microorganisms in milk, dairy products, egg, water, and other food materials (Qin, Chang, Barbosa‐Cfinovas, & Swanson, 1995). Sarkis et al. (2015) used the PEF and high voltage electrical discharge as the pretreatment in oil extraction from sesame seeds with the purpose of destructing the cell wall and increasing the oil extraction efficiency. Their results indicated that when the output energy of high voltage process increases, the destruction index might increase and also these two procedures lead to an increase in oil extraction efficiency. Tale Masouleh, Asadollahi, and Eshaghi (2015) used the PEF as the pretreatment for oil extraction from sesame seeds. Their findings revealed that PEF assists the procedure of oil extraction from sesame seeds with the use of cold pressing and an electric field with intensity of 1 kV/cm and pulse power equals to 50 might led to the maximum efficiency. La et al. (2016) used the PEF to produce fat from microalgae. They used the PEF with a low energy in their research and expressed that this procedure can be an appropriate replacement for common and usual methods of fat extraction.

To the best of the authors’ knowledge, there are no studies on the application of PEF and microwave as pretreatments in oil extraction from black cumin seeds. Therefore, the objective of the current research was to study the influence of applying novel technologies as the pretreatments in the oil extraction of black cumin seeds and their influence on the physicochemical properties and chemical composition of the final extracted oil.

## 2 MATERIALS AND METHODS

Black cumin seeds were provided from a local market in Gonbad‐e‐Kavoos (Golestan, Iran). Chemicals including ethanol, acetonitrile, phenolphthalein, sodium thiosulfate, chloroform, and hexane were purchased from Merck company (Germany). The equipments applied in the present research are as the following: laboratory sieve, laboratory oven (Memmert, Buchenbach, Germany), digital scale (Gec Avery, Smethwick, UK), microwave (LG, Seoul, South Korea), PEF device (made by Research Institute of Food Science and Technology, Mashhad, Iran), spectrophotometer (Biochrom, Cambridge, UK), kjeldahl (Auto Analyser 130 Tecator CO, Warrington, UK), Scanning Electron Microscopy (SEM) (S‐360, Oxford., England), refractometer (Abbe, Kobe, Jepan), Gas Chromatography/Mass Spectrometry (Agilent, San Francisco, USA), Rancimat (Metrohm, Herisau, Swiss) and laboratory screw press (Kern Kraft, Karlsruhe, Germany).

### 2.1 Sample preparation and oil extraction

Black cumin seeds (with 40.4% oil) were cleaned and kept in plastic bags resistant to air and moisture penetration by the time of the experiments. Two pretreatments of microwave with the power of 540 W for 180 s and PEF with 3.25 kV/cm electric field intensity and pulses equals to 30 were applied on seeds. It should be mentioned that to use of Molecular Weight (MW), the samples were located on the particular container of microwave in a row. The microwave power was set on 2,450 MH. In terms of PEF, the samples were put in chamber and distilled water was added on the samples as well. The applied power in chamber was 30 pulse with the width of 20 μs. Finally the samples were filtered and dried in 50°C. Then, the oil of seeds was extracted by a screw press with 34 rpm rotation.

### 2.2 Determination of oil extraction efficiency

The efficiency of oil extraction was computed through the Equation 1 (Bakhshabadi et al., 2017).

(1) $$\text{Extraction efficiency}=\frac{\text{Oil extraction yield}}{\text{Total oil content}}\times 100$$

### 2.3 Measurement of oil physical properties

The refractive index of oil was detected by a refractometer at a temperature of 25°C according to AOCS Cc‐7‐25 (1993). To measure the density of the extracted oil, a pycnometer was used (AOCS Cs 10a‐25, 1993). Briefly, the weight of empty pycnometer was recorded (*m*_1_),the weight of the pycnometer with distilled water (*m*_2_) as well as the weight of pycnometer with oil (*m*_3_) were measured, then the oil density (kg/m^3^) has been calculated through the Equation 2.

(2) $$\text{density}=\frac{m_{3}-m_{1}}{m_{2}-m_{1}}\times 1000$$

For color analysis, the spectrophotometer and AOCS Cc 13‐92 method (1993) was applied. For this purpose, the optical density of the oil was measured at 460, 550, 620, and 670 nm with spectrophotometeric approach and then through Equation 3 and according to the Lovybond yellow color, the color index of oils was determined.

(3) $$\text{Color Index}=1.29A_{460}+69.7A_{550}+41.2A_{620}-56.4A_{670}$$

### 2.4 Determination of the oxidative stability index

The Rancimat device and the AOCS Cd12b‐92 method (1993) were applied to determine the oil stability against oxidation. In this experiment, the temperature of 110°C and the air flow rate of 25 L/hr were used.

### 2.5 Measurement of meal protein

The amount of nitrogen in seeds was measured with the use of the full‐automatic Kjeldahl device and according to the AOAC990‐03 method (2008) which consisted of three steps including digestion, distillation and finally titration. After titration, the amount of nitrogen was determined and the amount of protein was computed with the use of the conversion factor of 6.25.

### 2.6 Scanning electron microscopy (SEM) analysis

The microstructure of PEF‐pretreated and untreated samples were observed by SEM (S‐360, Oxford, UK) regarding the conditions that have been set for this equipment. The samples were fixed on the silicon wafer and coated with gold to avoid of charging under the electron.

### 2.7 Analysis of the chemical components in extracted oil

A Gas Chromatography/mass spectrometry (GC/MS) device and Adam's method (1995) with some modifications have been used to analyze and determine various chemical components which exist in the black cumin seed oil. For this reason, 1 ml of oil was mixed with dry sodium sulfate anhydrous and hexane. The obtained mixture analyzed with the use of the GC/MS device equipped with a (HP‐SMS, Calabria, Italy) and capillary column (30 m length, 0.25 μm diameter and 0.25 μm thickness of internal layer). The helium carrier gas flow was 0.8 ml per minute and the fission rate of the samples was 1–10. The column temperature program was set up from 60 to 260°C with a speed of 4°/min. The mass spectra were prepared at 70 electron volt and the range of these spectra were 35–350 m/z. Determination of oil components was performed as a result of comparing their mass spectrum with spectrum bank and comparing their retention coefficient with the reference values. The retention coefficients were prepared with the use of the retention times of normal alkanes that were injected with the same device and under chromatographic conditions. The relative values of components were computed on the basis of the total peak level by the device software.

### 2.8 Statistical analysis

This research was performed in the form of a simple random design with three replications and the achieved results were analyzed with the use of the SAS statistical software (9.3.1, New York, USA). Comparison of data means was performed with the use of Duncan's multirange test with the confidence level of 95%.

## 3 RESULTS AND DISCUSSION

### 3.1 Efficiency of oil extraction process after pretreatments with PEF and microwave

**Figure 1:**
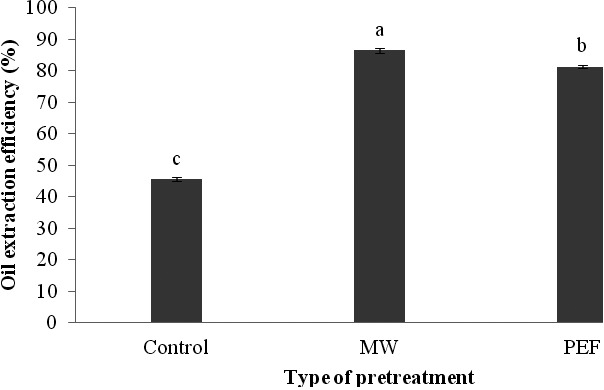
The influence of various pretreatments on the efficiency of oil extraction process from black cumin seed

**Figure 2:**
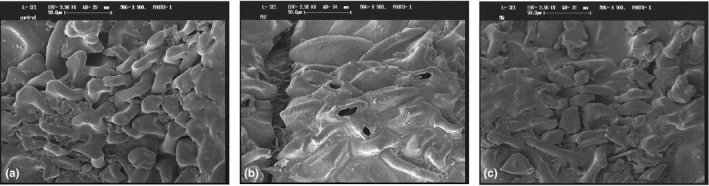
SEM micrographs of the control sample (a), the treated sample with pulsed electric fields (b) and the treated sample with MW (c)

Comparison of data means, using Duncan's test indicated that use of PEF and microwave pretreatments in this research led to an increase in the efficiency of oil extraction process (Figure 1). Also, it was specified that those samples pretreated by microwave had a higher oil extraction efficiency in comparison with other samples. This could be attributed to the increased fracture of cell walls which contain oil during treatment with microwave (Uquiche, Jeréz, & Ort, 2008). Also, Mohamed and Awatif (1998) reported that the higher efficiency of oil extraction through heating with microwave may be resulted from destruction of protein components. Our findings conformed to the results of Momeny et al. (2012), and Nde, Boldor, and Astete (2015). On the other hand, Schroeder, Buckow, and Knoerzer (2009) expressed that applying PEF leads to an improvement in extraction efficiency because of electrical decomposition of cells and their higher permeability. Our results were in agreement with the results of Guderjan, Topfl, Angersbach, and Knorr (2005).The morphological analysis by Scanning Electron Microscopy (Figure 2) confirmed that application of PEF and microwave pretreatments will lead to disintegration of cells and consequently better extraction of oil from them.

### 3.2 Influence of PEF and microwave pretreatments on physical properties of black cumin seed oil

#### 3.2.1 Refractive index

**Figure 3:**
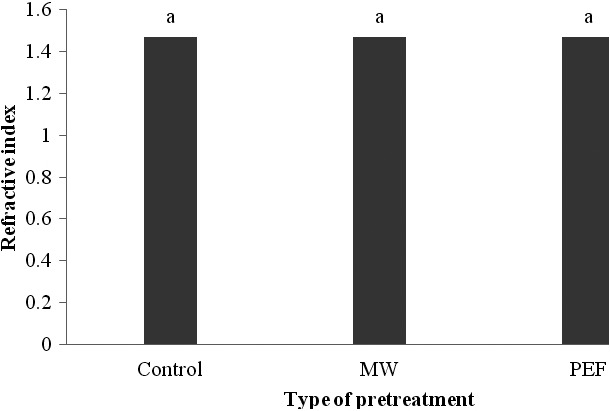
The influence of various pretreatments on the refractive index of black cumin seed oil

The refractive index is usually used as a criterion for oil purity. This parameter will increase with higher chain length (however the relation is not a linear one) and the nonsaturation level. Different oils and fats have their own and particular refractive indices, so this feature is used to identify and determine the purity of oils and fats (Bakhshabadi et al., 2017). The refractive index is useful in controlling the progress of reactions such as hydrogenation and catalytic isomerization of oils. Also, it is used to recognize the oil oxidation too, and the temperature and saturation are some factors that influence the refractive index (Malek, 2001).As shown in Figure 3, applying various pretreatments didn't have any significant influence on the refractive index of black cumin oil (*p*>.05) and for all samples about 1.469. Haroon et al. (2014) stated that based on the variety, the refractive index of black cumin oil is ranged from 1.4697 to 1.4730, which conformed with our results. Bakhshabadi et al. (2017) attributed no changes in refractive index of oils obtained from microwave process to the similarity of fatty acid compositions in the nontreated and treated samples. Also, Gorji et al. (2016) through studying the physicochemical properties of orange seed oil extracted by various methods, indicated that modern extraction technologies in comparison with common and usual methods, do not change physical properties of oils such as refractive index.

#### 3.2.2 Oil density

**Figure 4:**
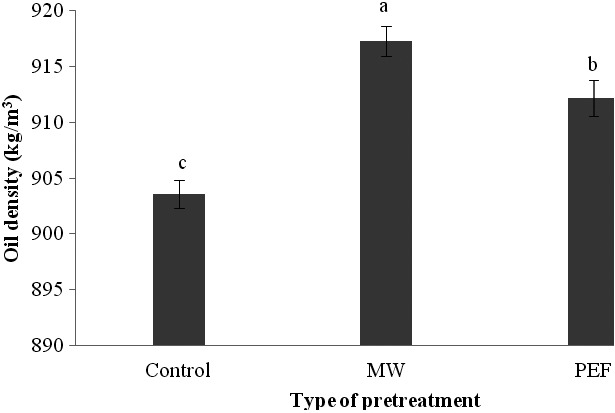
The influence of various pretreatments on the oil density of black cumin seed

It was found that different pretreatments in oil extraction from black cumin led to an increase in oil density (Figure 4). When the microwave and PEF were used, the oil density was increased 1.51% and 0.96%, respectively in comparison with samples that didn't have any pretreatments. Likely, this increase can be attributed to the more decomposition of seed cell walls and increase in resulted particles within the oil. Anjum, Anwar, Jamil, and Iqbal (2006) reported that microwave leads to a higher oil density that was inconsistent with the findings of Uquiche et al. (2008) and Kittiphoom et al. (2015). Theoretically, density is not an important property but economically it is very important. Although the oil shipments are sold on the basis of their weight but they are measured on the basis of their volume and also their relationship is defined by density. This parameter is not similar for all the oils and it is dependent on the composition of fatty acids, the trace and low compounds that exist in oil, and also the temperature (Hamm, Hamilton, & Calliauw, 2013).

#### 3.2.3 The color index of extracted oils

**Figure 5:**
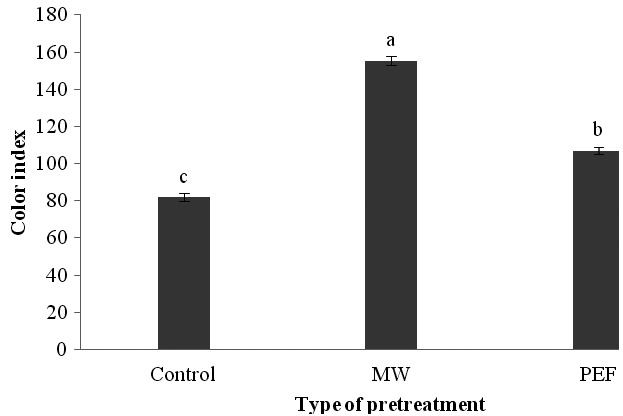
The influence of different pretreatments on the color index of black cumin seed oil

Our analysis of variance indicated that the influence of pretreatment type on the color index of oils extracted from black cumin seeds was completely significant (*p*<.01). Comparing the means of data obtained from samples (Figure 5) revealed that the color index ranged between 82.1–155.34. As it is clear, the use of PEF and microwave pretreatments caused an increase in the color index of black cumin oil and the maximum color index was obtained for microwave pretreatment. The increase in color index can be attributed to the fracture of plant tissues during treatment and therefore more extraction of the pigments. In this regard, Lee, Oh, Chang, and Kim (2004) studied the influence of different temperatures of parching the safflower seeds on its oil color. They stated that formation of color in oil was under the influence of parching temperatures in such a manner that when the temperature is increased, the oil color would change from light yellow to dark brown. Also, Kim et al. (2002) reported a distinct color increase in the rice bran oil at higher parching temperatures. Megahad (2001) attributed the increase in color in peanut oil as the result of treating the seeds with microwave to the nonenzymatic browning reactions and destruction of phospholipids during the parching process; so an increase in the brown components can be occurred together with elevation of other components as a result of decomposition of phospholipids. Guderjan, Elez‐Martínez, and Knorr (2007) observed similar results at the time of using PEF.

### 3.3 Influence of PEF and microwave pretreatments on the oxidative stability of produced oils

**Figure 6:**
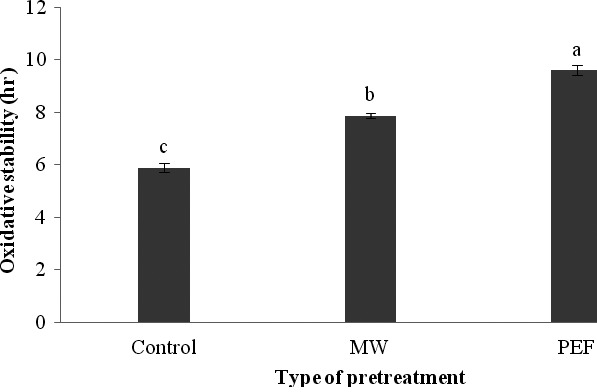
The influence of different pretreatments on the oxidative stability of black cumin seed oil

Oxidative stability is the time required to reach a point in which one oxidation index such as peroxide value or carbonyl number increases suddenly and it causes an undesirable taste and aroma in the oil. There are several methods to evaluate the chemicals resulted from heating processes which have so many influences on the quality and nutritional properties of oils, and the oxidative stability index is the most important one (Holser, 2003). Measurement of oxidative stability index alone during the heating processes of oils is not sufficient to evaluate the quality of oils, but it gives some information about the initial situation of oil sample (Matthaus, 2006). As can be seen in Figure 6, our results indicated that PEF and microwave pretreatments increased the oxidative stability of black cumin seed oils; the highest oxidative stability obtained with PEF treatment and then, with the microwave pretreatment. This can be explained by the increase in tocopherols and other antioxidant components in extracted oils as a result of these pretreatments. More details will be provided later in section 3.5. Spielmeyer, Wagner, and Jahreis (2009) and Guderjan et al. (2007)found similar results and claimed that use of different pretreatments leads to an increase in oxidative stability of extracted oils.

### 3.4 The meal protein of black cumin seeds

**Figure 7:**
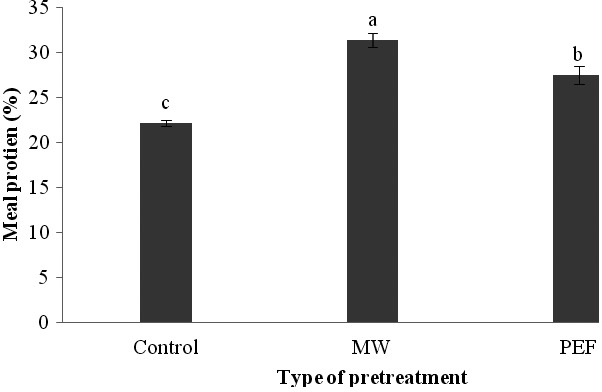
The influence of different pretreatments on the amount of protein in the meals of black cumin seed

The optimum use of required protein for body is dependent to the digestibility and pattern of essential amino acids in the foods(Taghizadeh, Asemi, Shaker Hoseini, Aminpour, & Valaiee, 2007). On the other hand, pricing the meals in the oil extraction companies is performed on the basis of their protein and moisture level (Rostami, Farzaneh, Boujmehrani, Mohammadi, & Bakhshabadi, 2014).We found that the influence of PEF and microwave pretreatment on the amount of protein in meals was completely significant (*p*<.01). Comparing the means of data (Figure 7) indicated that the maximum and minimum protein levels were related to the samples treated by microwave and the control, respectively. In other words, use of PEF and microwave treatments in oil extraction caused an increase in the protein of meal compared with the sample which didn't have any pretreatments. The reason of this phenomenon can be attributed to the extraction of more oil from the seeds and consequently, increase in the percentage of remaining proteins in the meals. Choi, Choi, Chun, and Moon (2006) and Sarkis et al. (2015) reported an increase in the amount of extracted protein by the microwave and PEF methods, respectively.

### 3.5 The influence of different pretreatments on the chemical compositions of oils

Our results revealed that the influence of PEF and microwave pretreatments on the amount of chemical components of oil samples were quite significant (*p*<.01). As it is clear in Table 1, in all samples, the amount of thymoquinone was higher than other components and this is the dominant phenolic component in the black cumin oil. Thymoquinone has probably some antioxidant, anti‐inflammatory, and anti‐cancer properties and can inhibit and control the oxidative stresses (Khan & Sharma, 2003). According to Table 1, it can be stated that when PEF was used as a pretreatment, much more components were extracted into the oil. After thymoquinone, cymene was the main component and it was completely conformed to the results of El Dakhakhny, Barakat, Abd El Halim, and Aly (2000). Some of these components were not observed in the control samples without any treatments; probably these components remain in the seed but when the microwave or PEF pretreatments were used, these components found their way into the oil.

**Table 1:** The influence of different pretreatments on the chemical composition of black cumin seed oil

Compound	Treatment		
	Control	MW	PEF
Cymeme	0.71^c^	1.34^b^	1.94^a^
Thymoquinone	1.02^c^	2.06^b^	2.57^a^
Cyclopropanemethanol, α., 2‐di methyl‐2‐(4‐methyl‐3‐pentenyl)	0.02^b^	0.37^a^	0.36^a^
2‐Amino‐4,6‐dihydroxypyrimidine,N,O,O′‐tris(trifluoroacetyl)	0.00^c^	0.11^a^	0.08^b^
Cyclohexanol	0.35^c^	1.30^a^	1.10^b^
Chlorine compounds	0.35^c^	0.96^a^	0.75^b^
β‐Pinene	0.00^c^	0.10^b^	0.14^a^
D‐Limonene	0.00^c^	0.09^b^	0.12^a^
Longifolene	0.00^c^	0.10^b^	0.17^a^
γ‐Terpinene	0.00^b^	0.00^b^	0.21^a^

Numbers with different letters in each row imply significant differences in the 5% level of probability.

PEF, pulsed electric fields.

## 4 CONCLUSION

The results of this research indicated that application of microwave and PEF leads to an increase in the efficiency of oil extraction process, oil density, oxidative stability, the color index of oil, and protein of the meal. Also, our results revealed that different pretreatments didn't have any significant influence on the refractive index of the oils. On the basis of results obtained from this study it can be said that applying microwave and PEF for treating the black cumin seeds before oil extraction by the cold pressing was effective in improve the qualitative and quantitative properties of the extracted oil. Also, it was found that the main component in the black cumin oil was thymoquinone and then, cymene.

## CONFLICT OF INTEREST

None declared.
